# Optimization of use-wear detection and characterization on stone tool surfaces

**DOI:** 10.1038/s41598-021-03663-4

**Published:** 2021-12-17

**Authors:** Antony Borel, Raphaël Deltombe, Philippe Moreau, Thomas Ingicco, Maxence Bigerelle, Julie Marteau

**Affiliations:** 1 Histoire Naturelle de l’Homme Préhistorique, HNHP-UMR 7194 (MNHN, CNRS, UPVD), Alliance Sorbonne Université, Département Homme et Environnement, Muséum national d’histoire naturelle, 17 Place du Trocadéro, 75116 Paris, France; 2 Institute of Archeological Sciences, Eötvös Loránd University, Múzeum krt. 4/B, 1088 Budapest, Hungary; 3 Laboratoire d’Automatique, de Mécanique et d’Informatique industrielles et Humaines (LAMIH UMR-CNRS 8201), Université Polytechnique des Hauts de France, Le Mont Houy, 59313 Valenciennes Cedex 9, France; 4 Laboratoire Roberval, Sorbonne Université, Université de Technologie de Compiègne, Centre de Recherches de Royallieu, 60203 Compiègne, France

**Keywords:** Materials science, Archaeology

## Abstract

Debates and doubt around the interpretation of use-wear on stone tools called for the development of quantitative analysis of surfaces to complement the qualitative description of traces. Recently, a growing number of studies showed that prehistoric activities can be discriminated thanks to quantitative characterization of stone tools surface alteration due to use. However, stone tool surfaces are microscopically very heterogeneous and the calculated parameters may highly vary depending on the areas selected for measurement. Indeed, it may be impacted by the effects from the raw material topography and not from the altered zones only, if non-altered part of the surface is included in the measurement. We propose here to discuss this issue and present a workflow involving the use of masks to separate worn and unworn parts of the surface. Our results show that this step of extraction, together with suitable filtering, could have a high impact on the optimization of the detection and thus characterization of use traces. This represents the basis for future automatic routines allowing the detection, extraction and characterization of wear on stone tools.

## Introduction

The surface of the objects (stone tools in our case) is the most exposed element to external contact and is therefore most directly affected by each type of physical and chemical attack from its environment. Most of these attacks (either due to natural or human agents) modify surface topographies^1^. Thus, the observation and characterization of surface topographic signatures on stone tools can provide us with clues about the processes undergone. It is this principle that has been used and applied for a long time by wear analysts to interpret the function of prehistoric stone tools by describing qualitatively the surface alterations.

However, if traceology of prehistoric stone tools is to date an essential approach for understanding the archaeological artifacts’ function, an inherent part of subjectivity and uncertainty remains in its interpretations. Indeed, rare are traces that can qualitatively and exclusively be linked to only one specific activity. The interpretation of the use of a stone tool is only tentative from the combined analysis of different types of traces. This question of probability and its associated uncertainty is rarely acknowledged in the literature presenting the results of traceological studies. Also, a certain number of questions, already raised in the 1980s, related to the reliability and reproducibility of the method in particular, are still relevant today^2–8^. This is why the development and/or application of quantitative methods, which would complement the still fundamental qualitative approach, are essential for the future of the discipline.

First attempts at applying quantitative methods for wear identification dates back to the 1970s, with the seminal work of Semenov who measured the intensity of polishes and compared microreliefs between used and unused surfaces^9, 10^. Since, few studies have explored different avenues of quantification of stone tool surfaces e.g.^11–16^ using different acquisition techniques e.g.^17–20^. Since the 2000s, acquisition instruments have become more accessible and efficient allowing an increasing number of studies dedicated to quantitative analysis, surface texture analysis in particular. We reviewed 44 papers focusing on surface texture analysis of stone tools and published, mainly in international journals, from 1982 to 2021 (see references in Supplementary Table S1a and b). Most of the recent results are very promising, showing that metrological parameters can discriminate wear resulting from different use activities and worked materials. However, comparison of results is challenging, primarily because of a lack of standards for the discipline (see data in Supplementary Table S1a and b). A couple of very recent studies sought to improve the reproducibility of protocols and repeatability of results by testing the parameters of acquisition systems (e.g. the effect of numerical aperture of the objectives on the surface measurements^21^) or by proposing observation procedures^22^. However, different surface acquisition technologies are used by wear analysts such as confocal microscopy (N = 28/44; 64% of the reviewed papers), interferometric microscopy (N = 7/44; 16%), focus variation microscopy (N = 3/44; 7%) or atomic force microscopy (N = 3/44; 7%). In addition, measurements are still performed with different objectives, numerical aperture and size of scanned area, data are processed with different filters and the metrological parameters calculated are also variable. The need for standardization both in qualitative and quantitative wear analysis was already underlined few years ago^23^. At that time Macdonald^24^ already suggested to focus future research on the determination of the best area of analysis to test if measurements should be performed on areas where the wear is covering the full field of view or if unworn part of the surface should be included. Evans et al.^25^ also called for the production of “adequate algorithms that can isolate worn areas of surface from unmodified areas”. So far, what could be considered as the best area of analysis has not been tested and most of the studies follow a procedure in which rectangle areas filled with micropolish (or located on the most developed polish of the sample) are manually selected over the surface of the measured stone tool^26–33^. This type of selection provides promising results but also implies few limitations. For example, the choice of microscope objective and/or acquisition scale, and even sample, are sometimes restricted by the search of a field of view filled with polish.

We propose here to examine how the selected area for measurement can impact the detection and characterization of use-wear on stone tools in order to propose a method which could cope with known limitations and optimize surface texture analysis on stone tools.

## Results

In order to have a first insight about the relation between use and surface alteration of stone tools, we compared the commonly used arithmetic mean deviation parameter (Sa) of surface measurements before use and after 10,000 cycles of wood sawing. Two surfaces with qualitatively different levels of surface alteration were selected and the following ratio was computed:

1 $${S}_{a, ratio}= \frac{{S}_{a, \mathrm{10,000}\, cycles}-{S}_{a, 0\, cycle}}{{S}_{a, 0\, cycle}}x100$$

**Figure 1:**
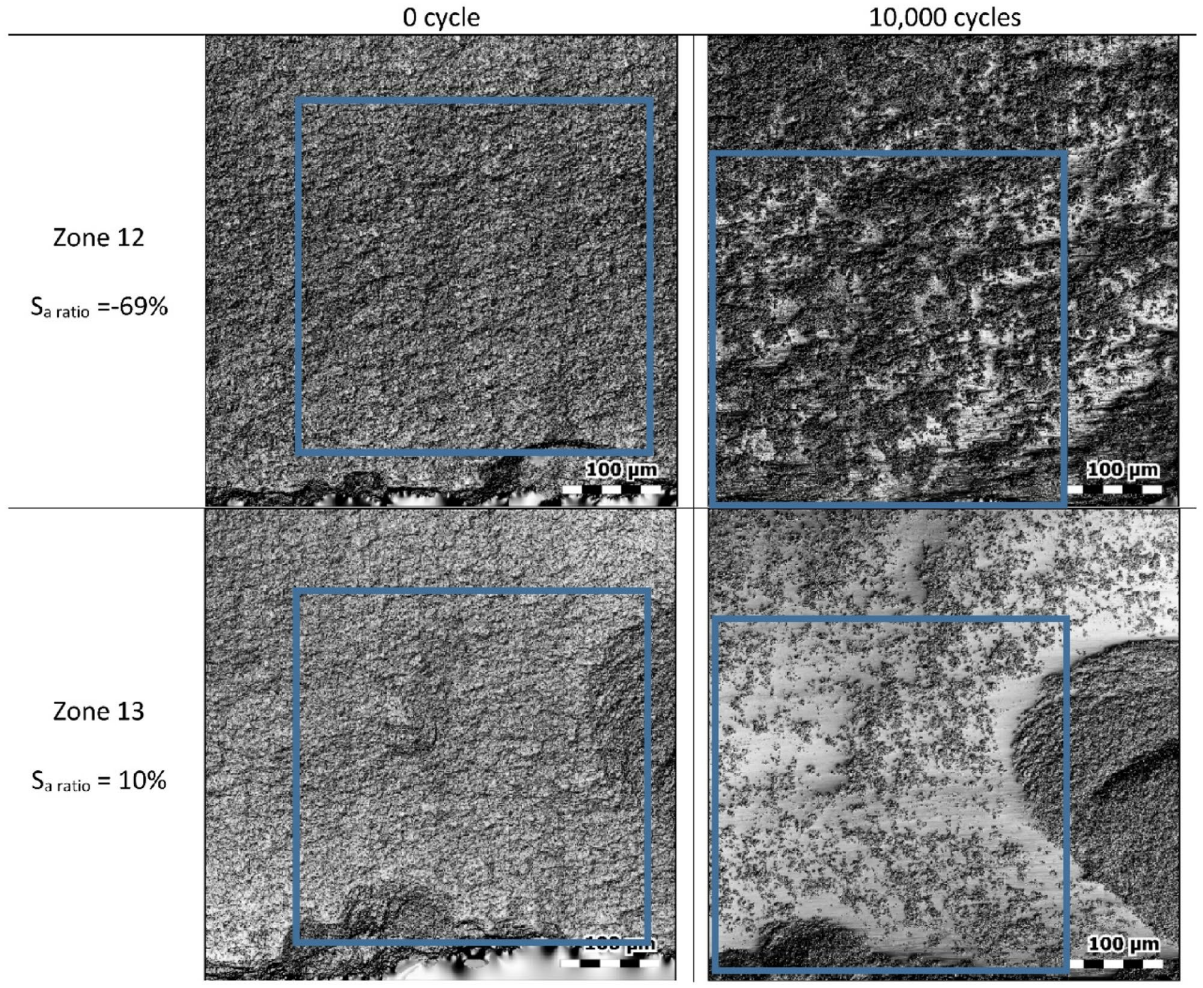
Photo simulation images of two locations and corresponding Sa ratios. Matching sub-surfaces are shown by the blue rectangles for each zone.

Figure 1 shows photo simulation images of the measurements performed on two selected zones before and after use, as well as the computed S_a_ ratios at full scale (i.e. without filtering). The use of photo simulation images highlights wear, which corresponds to whiter areas. Both locations are heterogeneously modified by wear: wear seems to be more pronounced in some locations. While use-wear is evident on each of these surfaces, very different Sa ratios were obtained for each area: negative and positive values of 69% and 10% are respectively found for zone 12 and zone 13. Four hypotheses can be proposed to explain these differences:

- The shifting of the zones prevents the detection of any wear signature with Sa ratios.
- The Sa values computed at full scale (i.e. without filtering) do not allow wear signature to be detected.
- The Sa is not a relevant roughness parameter for roughness description of these surfaces.
- The heterogeneity of the areas (comprising worn areas and unworn areas) prevents any wear trend detection.

The first hypothesis can be easily tested by selecting sub-surfaces that exactly match. These sub-surfaces are represented through the use of blue rectangles in Fig. 1. The new ratios computed for these sub-surfaces are equal to − 61% for zone 12 and − 2% for zone 13. There is thus a clear change of values, particularly when the Sa computation encompasses holes or severe roughness changes near the edge of the tool. However, it does not explain the Sa discrepancies between zone 12 and zone 13.

The second hypothesis was tested by computing Sa values on measurements from both sides of the tools before and after use (N = 2 × 30 surfaces; see Supplementary Table S2) at full scale and after high-pass filtering.

**Figure 2:**
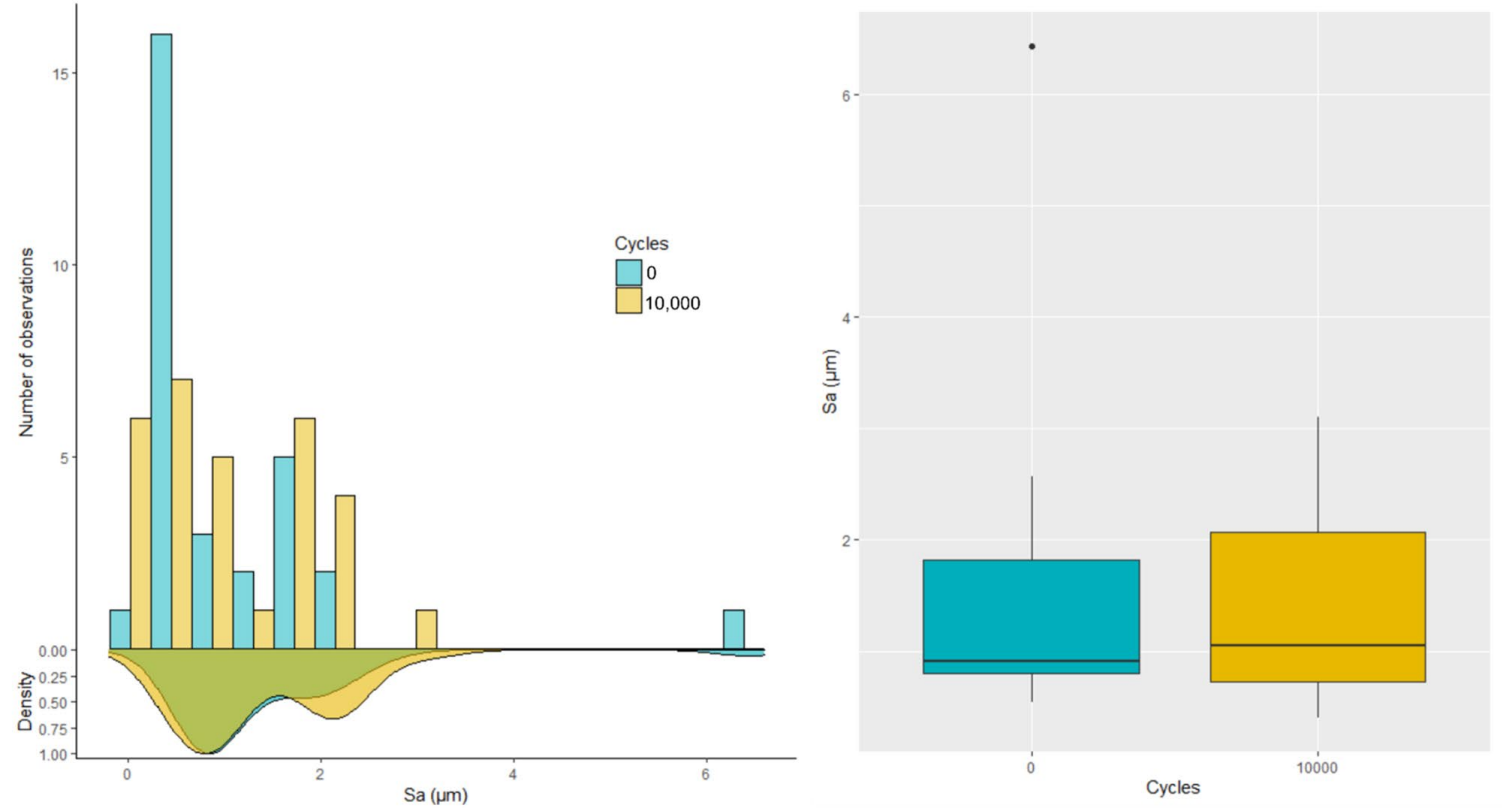
Histogram, density and boxplot of the arithmetic mean deviation (Sa) before (0 cycle) and after use (10,000 cycles). Sa values are computed for all the measurements closest to the edge, at full scale.

Figure 2 shows the Sa distribution at full scale. An average Sa of 1.4 µm with a standard deviation of 1.11 µm was found before tool use. After tool use, the Sa average was found to be 1.35 ± 0.76 µm. The computed Sa values before and after use showed large standard deviations and thus prevent any identification of wear effects on Sa values. No statistically significant difference of Sa can be noted between non-filtered surfaces before use and after 10,000 cycles (Wilcoxon signed-rank test: V = 244; p = 0.8236).

**Figure 3:**
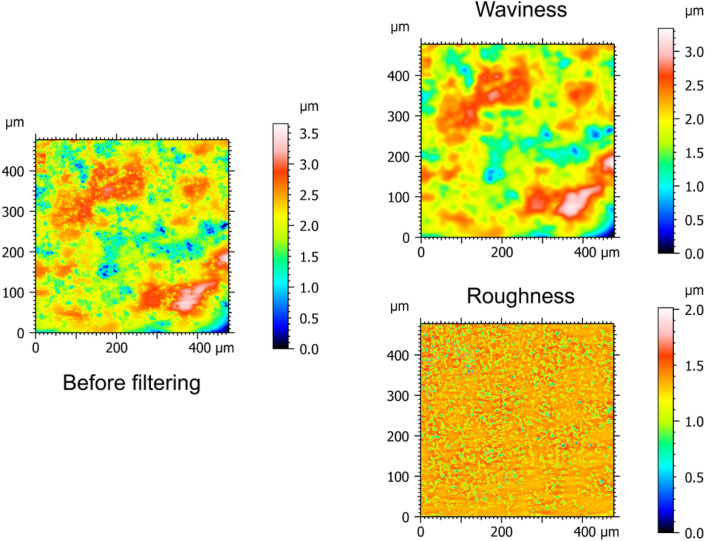
Example of filtering with Zone 12 (left) before filtering and (right) after filtering with a cut-off length of 25 µm: as a high-pass filter is used, only the roughness part is kept for the analysis.

**Figure 4:**
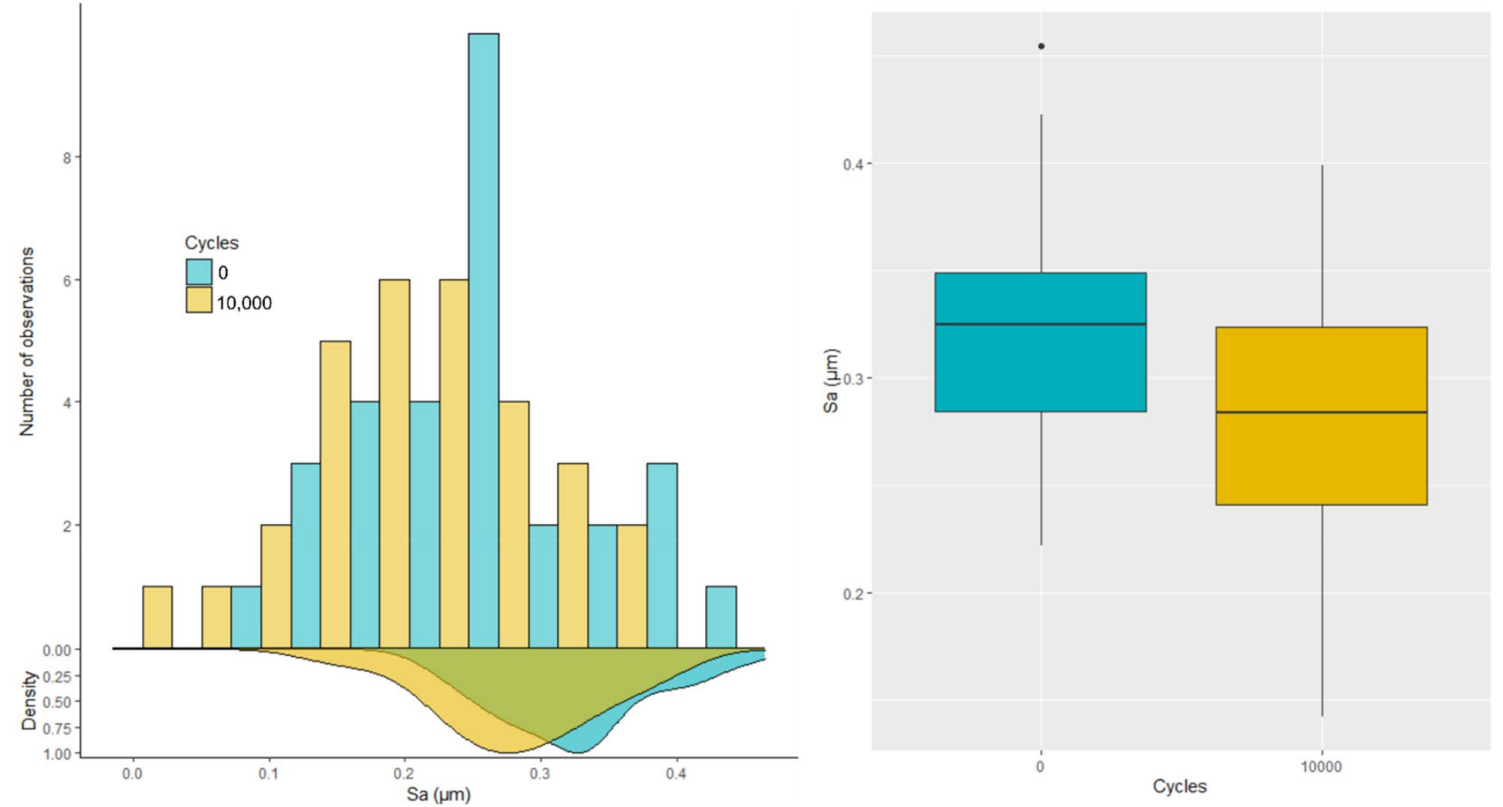
Histogram, density and boxplot of the arithmetic mean deviation Sa computed for all the measurements closest to the edge, after using a high-pass filter with a cut-off length of 25 µm.

To assess the influence of the scale, high-pass filtering was then performed on the measurements. High pass filtering was chosen to remove waviness and focus on microroughness changes. Through testing, it was found that the best results were obtained when using a Gaussian high-pass filter with a cut-off length of 25 µm^34^, as illustrated in Fig. 3. This step removes none relevant frequencies and allows focusing on relevant surface texture. Filtering was performed on all measurements from both sides of the tools (see Supplementary Table S2). Figure 4 shows the distribution of Sa values obtained for the filtered measurements before and after use. The average of the Sa values ± SD were found to be equal to 0.32 ± 0.06 µm before use and equal to 0.28 ± 0.06 µm after use. Filtering led to a considerable decrease of the average and spread compared to the results shown in Fig. 2. Moreover, statistically significant difference of Sa was found between filtered surfaces before use and after 10,000 cycles (Wilcoxon signed-rank test: V = 425; p = 0.000016). However, no discriminant relationship could be identified between wear, Sa values and cycles of use as the distributions still largely overlap (Fig. 4). Therefore, Sa values computed at full scale (i.e. without filtering) is not recommended to detect wear signature. Using high-pass filtering greatly improves surfaces differences detection but does not seem enough to properly discriminate wear signatures.

The third hypothesis was that the Sa, which is a commonly used roughness parameter, may not be the most relevant parameter for the detection of this type of wear (i.e. wear on stone tools but, here, wood sawing in particular). Other height parameters (Sq, Sp, Sv, Sz, Sku) as well as functional parameter (Smr, Smc, Sxp), and functional volume parameters (Vm, Vv, Vmp, Vmc, Vvc, Vvv) were computed. Before filtering, statistically significant differences are noted between surfaces before and after use for Sv (Wilcoxon signed-rank test: V = 388; p_Holm_ = 0.012) and Smr (V = 87; p_Holm_ = 0.026) only. After filtering, statistically significant differences were found for Sq (V = 392; p_Holm_ = 0.0042), Smc (V = 426; p_Holm_ = 0.00014), Sxp (V = 400; p_Holm_ = 0.0025), Vm (V = 437; p_Holm_ = 0.000039), Vv (V = 432; N = 30; p_Holm_ = 0.000066), Vmp (V = 437; p_Holm_ = 0.000039), Vmc (V = 398; p_Holm_ = 0.0028), Vvc (V = 434; p_Holm_ = 0.000053) and Vvv (V = 383; p_Holm_ = 0.0080). Again, filtering greatly increases differences but, like for Sa, the overlap between the values of these parameters for surfaces before use and after 10,000 cycles was found to be important. Therefore, none of them seem to be more relevant than Sa to discriminate wear signatures.

**Figure 5:**
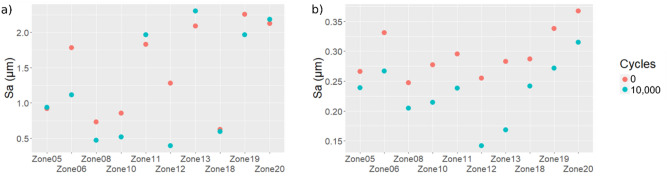
Arithmetic mean deviation (Sa) computed for the 10 selected measurements showing the largest wear traces (**a**) without filtering, (**b**) with filtering (high-pass filter with a 25 µm cut-off length).

It seems that the heterogeneity of the measured areas, comprising worn areas and unworn areas, within one measurement as well as between measurements, prevents any wear trend detection. To test this fourth hypothesis and better assess the effect of wear, ten locations (see Supplementary Fig. S3) visually showing the most extensive wear were chosen through the observations of the photo simulation images of the measurements. For these ten locations, the Sa was computed before and after use, with and without filtering (Fig. 5; see Supplementary Table S4).

**Figure 6:**
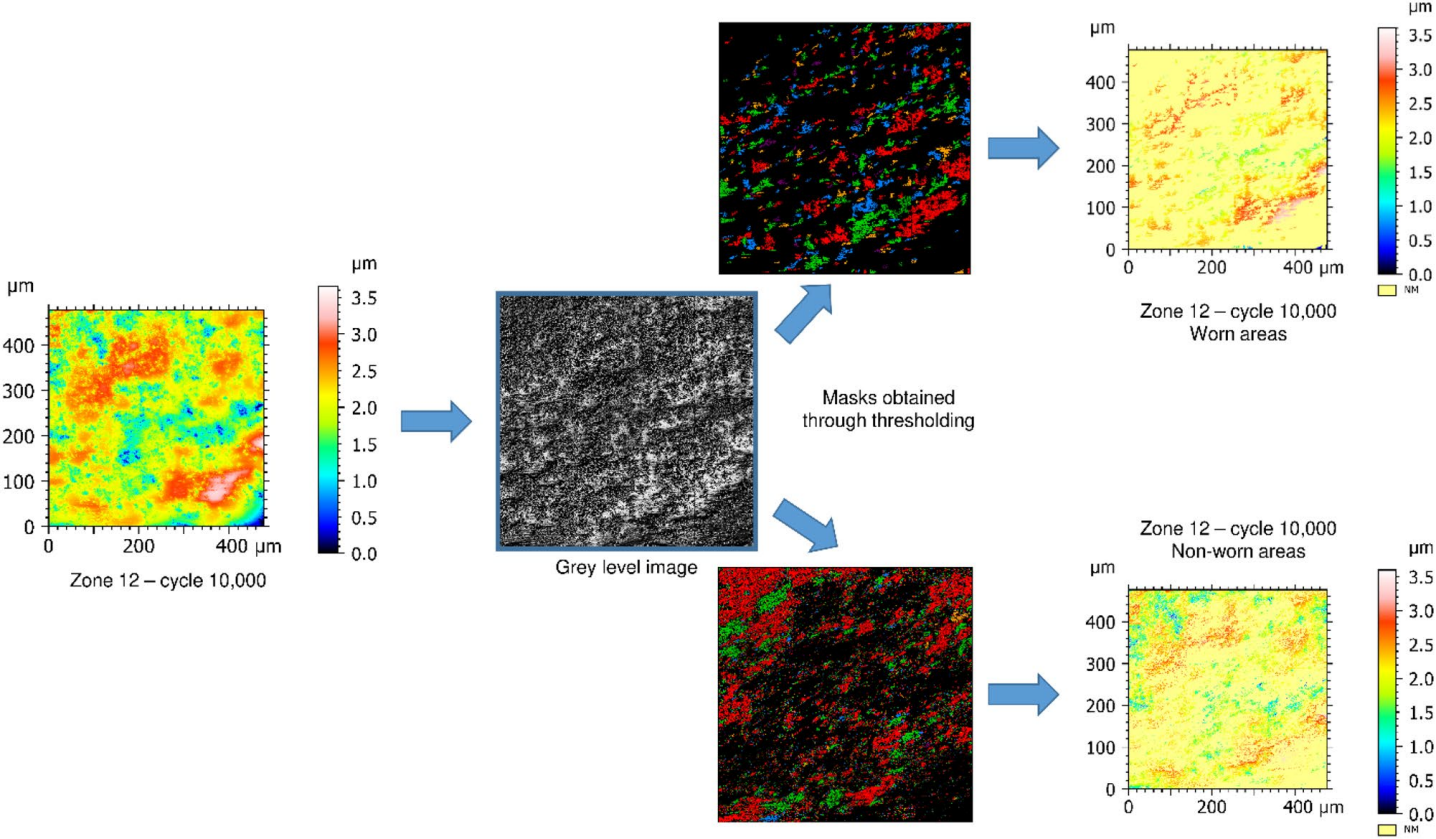
Methodology description to separate worn areas from non-worn areas. Manual tresholding is applied on the grey level distribution of the photo simulation image in order to control the selection of the worn and unworn surface and create corresponding masks. Then, masks are applied on the height map to produce two datasets: one containing data of the worn part of the surface and another one with the data of the unworn part. Missing data (NM) are displayed in yellow in the final surfaces.

Again, no trend can be identified without filtering (Fig. 5a). However, with filtering (Fig. 5b), a clear decrease of the Sa values can be observed for each zone (if considered separately). Similar tendency is observed on each zone for parameters Smc, Sq, Vv and Vvc (Supplementary Fig. S5). Based on the grey level distribution of the photo simulation images of these 10 locations, the worn areas were assessed to represent between 10 and 50% of the total area of the given measurement. The Sa computed at the relevant scale is thus a roughness parameter that is meaningful for wear examination when the initial surface (i.e. the surface before wear) is available for comparison with the worn one. However, with archaeological stone tools, the initial surface is never available for measurement. Therefore, a comparison of the Sa values before and after use is not possible. Moreover, so far, the selected areas comprise both worn and unworn parts. This may blur the wear signature. To overcome this issue, a new methodology was developed and is illustrated in Fig. 6.

**Figure 7:**
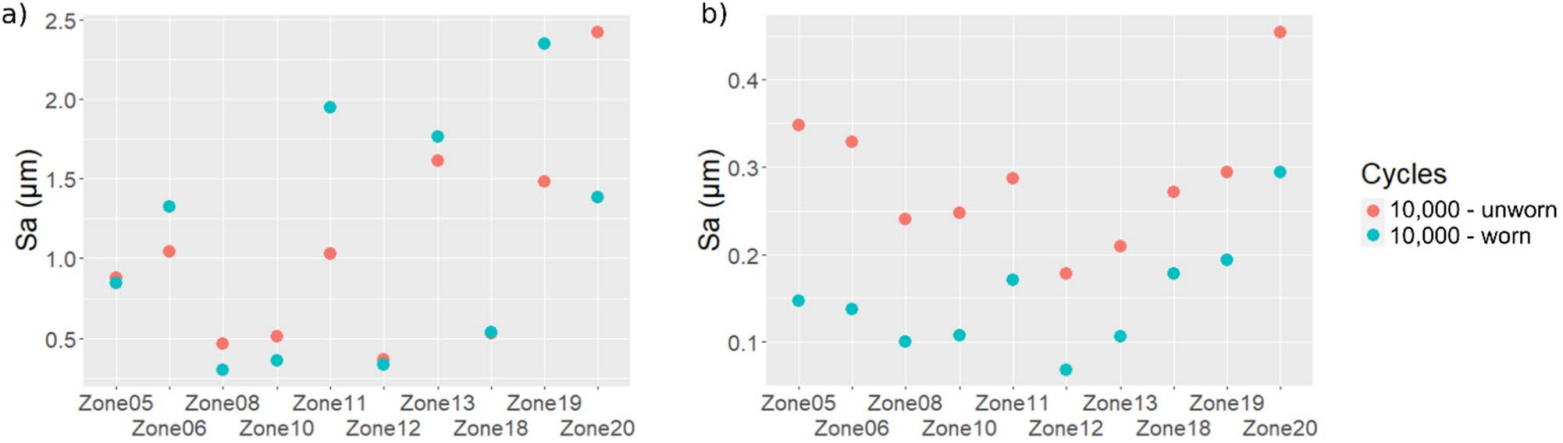
Arithmetic mean deviation (Sa) computed for the 10 selected measurements showing the largest wear traces. The Sa values are computed following the proposed methodology using masks (**a**) without filtering and (**b**) with filtering (high-pass filter with a 25 µm cut-off length).

First, photo image simulation is used to convert the examined measurement into a grey level image. Then, the distribution of grey levels is examined and thresholded to create masks. One mask is used to keep the measurement points corresponding to the worn areas only while the second mask is used to retain the measurement points corresponding to the unworn areas (i.e. the complement). Isolated points of the masks are removed to decrease possible noise. Finally, the Sa parameter is computed for each masked surface on unworn and worn parts after 10,000 cycles of use (see Supplementary Table S4). The results are shown in Fig. 7a without filtering and Fig. 7b with filtering. Again, no clear trends are obtained with the computation of the Sa parameter at full scale. However, after filtering, a clear decrease of the Sa values is observed for each examined location considered independently. Similar tendency is observed on each zone for parameters Smc, Sq, Sxp, Sz, Vmc, Vv and Vvc (Supplementary Fig. S6).

**Figure 8:**
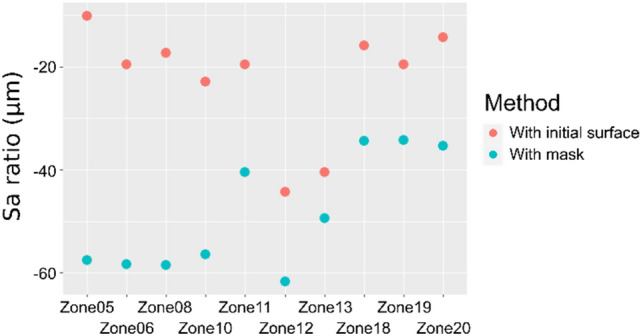
Comparison of Sa ratios computed with access to the initial surfaces versus with the proposed methodology using masks.

To compare the results given by this methodology (Fig. 7) with the results obtained with the surface before use (Fig. 5), Sa ratios are computed and shown in Fig. 8. The Sa ratios were computed using Eq. 1 for the computations made with the initial surface and using the following equation for the results obtained with the new methodology:

2 $${S}_{a, ratio}= \frac{{S}_{a, \mathrm{10,000}\, cycles\, (worn\, areas)}-{S}_{a, \mathrm{10,000}\, cycles\, (unworn\, areas)}}{{S}_{a,\mathrm{10,000}\, cycles\, (unworn\, areas)}}x100$$

Figure 8 shows that there is a global decrease of the Sa ratios with the proposed methodology. Therefore, using masks maximizes the difference between unworn and worn surfaces.

## Discussion

The experimentation presented here and the provided examples show that signatures could be quantitatively undetectable, even if they are qualitatively evident. This confirms that the integration of both qualitative and quantitative approaches in use-wear analysis is critical. Their complementarity, already stated among others by Macdonald^24^ and Ibáñez and Mazzucco^33^, is now “quantitatively” demonstrated here. Our results also show that, with such heterogeneous surfaces as those of stone tools, a slight modification of the measured area can have a high impact on the values of the calculated parameters. This reinforces the fact that the selection of the measured area with a visual control of the micrographs of the used surface is essential and remains, so far, the best procedure to follow. However, this selection usually corresponds to the field of view of the objective used for the surface acquisition or to rectangle subareas created in the analysis software^26–33^ (Supplementary Table S1a). This procedure faces limitations. Indeed, depending on the extent of the micropolish to be measured, selected areas have to be reduced in size to make sure it is filled with the worn surface^29, 31^. The use of masks to separate worn from unworn parts of the surface do not limit the size of the analyzed area and can also be applied on stitched datasets. Larger, more representative, areas that are considered as one of the keys for good classification of traces by Ibáñez and al.^29^ could thus be analyzed. Also, it should be noted that, even when wear impacted a small portion of the measured surface (around 10%), it was clearly detected with this methodology. Searching for an area where polish is sufficiently developed over the surface to fill the field of view and/or subareas of analysis is thus not necessary and this method could help characterizing polishes even at an early stage of development^33^. Therefore, it provides more flexibility to the analysts to find good combination of magnification, numerical aperture and working distance to obtain relevant measurements on samples (i.e. stone tools) whose uneven shapes already limit the observation^35^. Our results show an improvement of the differentiation between unworn and worn surface when masks are used. This improvement is likely to be found also when trying to differentiate polishes due to different use activities or contact materials. By maximizing the differences, masks might also improve the number of correctly classified types of wear by machine learning algorithms. Recent studies provided mixed results of classification of contact material^29, 32, 33^. In these studies, good classifications are often under 70%. These results are promising but still need improvement before applying such method on archaeological material. The use of masks is a way of possible improvement to be explored in future research.

Our study, like Ibáñez et al.^29^, shows the importance of filtering to analyze stone tool surfaces. It demonstrates that, whatever the mode of selection of the area of analysis, using filtering significantly improves the detection of wear. However, in order to determine and apply the most suitable filters, more experiments with different kind of contact materials and tasks need to be performed in the future. Filtering of stone tool surfaces has not been the focus of any paper so far but the already published studies involving surface texture analysis show that different filters are applied depending on the raw material of the tool, the activities carried out in the experiment and the signatures intended to be found (see Supplementary Table S1a and b).

Comparing the different parameters which can be used for surface roughness analysis is out of the scope of this paper. However, we have seen that, after filtering, Sa could be relevant for roughness description of stone tool surfaces and that the use of masks largely improves its capacity to differentiate worn from unworn areas. As shown in Supplementary Fig. S6, this is also the case for Smc, Sq, Sxp, Sz, Vmc, Vv and Vvc. In our example, the other parameters were not able to highlight differences between worn and unworn flint surfaces, even after filtering and area selection with masks, while for example an increase of Sku values with the increasing degree of wear was noticed by Pfleging et al.^36^ and Sp was among the parameters allowing a relatively good classification of cereal polishes in Ibáñez et al.^28^. Further research should focus on the behaviors of the parameters from ISO 25178^37^ when applied to the different use traces on stone tools. Also, evaluating their variability depending on the raw material of the tool and the different types of observed traces will allow a better classification model to be built. One drawback of using masks is that it excludes some roughness parameters from the analysis. Indeed, using masks creates missing data points in the data set and parameters such as Sal, Str or Sdq are not compatible with it. Masks should not be applied in studies intending to characterize traces which are found to be better described with such parameters.

To evaluate the evolution of the alterations, comparing surfaces before and after use seems an adequate solution to address the issue of the heterogeneity of stone tool surfaces. In experimental conditions, the initial surfaces (i.e. before use) of the stone tools are available if the artefact was measured before use but this is not the case for the archaeological artefacts. As a result, such comparison is not applicable to archaeological pieces. Comparing Sa ratios both for initial surfaces versus surfaces after use and for the proposed methodology using masks, we have shown that it is possible to identify similar patterns. This result demonstrates that applying masks is a relevant option to overcome the heterogeneity of stone tool surfaces and to develop a methodology applicable to archaeological samples.

We proposed here manual thresholding of the grey level distribution of photo simulation images in order to extract the worn and the unworn areas from the surfaces. Therefore, the analyst adjusts the mask selection based on the qualitative appreciation of the images. This procedure still keeps the inherent subjectivity of the analyst and may limit the reproducibility of the results and/or introduce biases in the surface comparisons. However, this issue can be partly overcome by providing the original surface texture dataset together with all the settings used for the photo simulation and for thresholding. With these data, any wear analyst can obtain the same masks and repeat the analysis. The validation of the use of masks for the optimization of surface texture analysis of stone tools and the possibility, offered by this method, to reproduce the analyses once the surface is acquired represent an important step forward towards the automation of lithic use-wear quantification^25^.

## Conclusion

The procedure of selection of the best areas of analysis is a key aspect of surface texture analysis on stone tools. Indeed, the heterogeneity of use-wear on stone surfaces complicates their observation and measurement. Our results demonstrate that the use of masks to separate worn and unworn parts of stone surfaces maximizes the differences and thus allow better detection and characterization of use-wear. It has the disadvantage of excluding few parameters (e.g. Sal, Str, Sdq) which are not compatible with missing values in the surface texture dataset. Nevertheless, apart from enhancing wear characterization, it provides more flexibility in the choice of the acquisition configuration (i.e. objectives, location of the area of interest, state of development of micropolish), the extraction of the worn area is possible and the results are fully reproducible (given that the original dataset of the surface and few settings are provided). In order to pursue the methodological development of automated quantitative analysis, further research will investigate if the optimum parameters for the photo simulation generation and for thresholding can be automatically and reliably computed based on the stone tools surface properties.

## Methods

### Sample and experiment

Flint (Jurassic flint from Kremenets', Ukraine) was chosen to carry out the experiment as traces on this type of raw material are now qualitatively well-known among wear analysts. The used flake measured 84.9 mm in length, 35.8 mm in width and had a maximal thickness of 7.96 mm (see Supplementary Fig. S7). The use experiment was performed with the tribometer Bruker UMT TriboLab equipped with a 100 N force sensor. In order to hold the flake and to fix it on the force sensor, a made-to-measure support has been designed with CAD software (Catia) and 3D printed in Polylactic acid (PLA) using an Ultimaker S5 printer. The flake was clamped into the support by pouring acrylic resin (Clarocit Struers) around it.

Once attached firmly into the tribometer, the flake was used to saw a 18 cm diameter piece of fresh wood (*Corylus avellana*) with bark, hold on the bottom of the tribometer also by a made-to-measure 3D printed support (see Supplementary Fig. S7). The speed of the sawing movement was setup at 20 mm/s with a constant force of 10 N. Ten millimeters of the edge of the flake was used; i.e. was in contact with the wood during the sawing activity. The maximum number of sawing cycles, which is the maximum number of back-and-forth movements, was equal to 10,000.

After the sawing cycles came to an end, the flint was cleaned in a 10% solution of Panreac Derquim LM02 phosphate free neutral detergent in an ultra-sonic bath for 20 min. Then, an ultra-sonic bath of 3.5% of hydrogen peroxide (35%) was performed for 20 min. Finally, the flake was cleaned with pure ethanol before observation to remove any potential remaining fatty residues.

### Data acquisition

In order to document the evolution of the signatures through time/cycles of use, the surfaces of the flint were measured before use and after 10,000 sawing cycles. Topography measurements were performed using a white-light interferometer (Zygo Newview™ 7300, Zygo Corp., USA) with a 50× / 0.55NA objective (working distance of 3.4 mm). The lateral resolution was equal to 0.52 µm and the vertical accuracy was about 1 nm. Stitching was used to obtain measurement areas equal to 477 µm × 477 µm with a step of 0.219 µm.

Both faces of the flake were documented on the 10 used millimeters and up to 1,05 mm from the edge. The measurements of the flake surface were performed before and after use thus giving a total of 248 surfaces. However, it was found that there was a slight shift of the measurement position of the tool before use and after 10,000 cycles. A careful examination of the surface features was used to identify the shift value and led to the exclusion of 10 measurements for both tool sides. Finally, for this study, we chose to only focus on measurements closest to the edge from both sides of the tool before and after use (N = 2 × 30 files) to clarify our findings and methodology.

### Data processing and statistical procedure

All the measurements were leveled using a polynomial of degree 3 to flatten out the surfaces (removal of form). When the procedure implied filtering, we applied a Gaussian high-pass filter with a cut-off length of 25 µm^34^ which provided the best results through testing. This filtering removes non-relevant frequencies and allows focusing on relevant surface texture. The proposed study mainly discusses the results of one roughness parameter: the arithmetic mean deviation Sa (ISO 25178^37^), an amplitude parameter which has been found to be the less sensitive to noise and outliers in previous study on stone tool^36^. Sa has also the advantage to be easily intelligible and a decrease of its value is expected with the increase of wear. This parameter thus seemed particularly appropriate to present and examine the method we propose here. However, 14 other roughness parameters were also tested: amplitude parameters (Sq, Sp, Sv, Sz, Sku), functional parameters (Smr, Smc, Sxp), functional volume parameters (Vm, Vv, Vmp, Vmc, Vvc, Vvv). The choice to exclude spatial parameters such as the autocorrelation length Sal, the texture aspect ratio Str or else hybrid parameters such as the surface slope Sdq was made to ensure a proper comparison of the proposed methodologies. Only roughness parameters withstanding missing data points were kept (i.e. we kept only parameters compatible with mask).

All parameters were computed using MountainsMap 7.4 software from Digital Surf. This software was also used to simulate photo images using the topography measurement. The horizontal lighting angle (alpha) was set at 130°, the vertical lighting angle (beta) was set at 30°, the contrast was equal to 8 and the intensity was set at 100%. Statistical tests, ratios and charts were computed in R v4.1.0^38^ through RStudio v1.4.1106. The used libraries were: tidyverse v1.3.1^39^, readxl v1.3.1^40^, ggpubr v0.4.0^41^ and stats v4.1.0^38^. The normality of the distributions of the parameters was tested using Shapiro–Wilk normality test (shapiro.test function). The results showed that, after filtering, the distributions were normal. However, the null hypothesis of normality of distribution could not be rejected for most of the parameters before filtering. Thus, non-parametric two-tailed Wilcoxon signed-rank exact test for paired sample (wilcox.test function) was used to compare the parameters mean of worn and unworn surfaces before and after filtering and with and without mask, always with N = 30. An alpha of 0.05 is used for every tests. We applied the Holm correction for multiple testing when necessary. Such adjusted p-values are indicated as “p_Holm_”.

The R code used for the analysis and to generate HTML report with the libraries knitr v1.33^42–44^ and rmarkdown v2.9^45–47^ is available in Supplementary Material S8.

## Supplementary Information

Supplementary Information 1.

Supplementary Information 2.

Supplementary Information 3.

Supplementary Information 4.

Supplementary Information 5.

Supplementary Information 6.

Supplementary Information 7.

Supplementary Information 8.

## Data Availability

Data used in the paper are provided in Supplementary materials or available on demand to the corresponding author.
