# Author Correction: Optimization of use-wear detection and characterization on stone tool surfaces

**DOI:** 10.1038/s41598-022-20545-5

**Published:** 2022-09-20

**Authors:** Antony Borel, Raphaël Deltombe, Philippe Moreau, Thomas Ingicco, Maxence Bigerelle, Julie Marteau

**Affiliations:** 1 Histoire Naturelle de l’Homme Préhistorique, HNHP-UMR 7194 (MNHN, CNRS, UPVD), Alliance Sorbonne Université, Département Homme et Environnement, Muséum national d’histoire naturelle, 17 Place du Trocadéro, 75116 Paris, France; 2 Institute of Archeological Sciences, Eötvös Loránd University, Múzeum krt. 4/B, 1088 Budapest, Hungary; 3 Laboratoire d’Automatique, de Mécanique et d’Informatique industrielles et Humaines (LAMIH UMR-CNRS 8201), Université Polytechnique des Hauts de France, Le Mont Houy, 59313 Valenciennes Cedex 9, France; 4 Laboratoire Roberval, Sorbonne Université, Université de Technologie de Compiègne, Centre de Recherches de Royallieu, 60203 Compiègne, France

Correction to: *Scientific Reports* https://doi.org/10.1038/s41598-021-03663-4, published online 17 December 2021

The original version of this Article contained errors in the spelling of the authors Antony Borel, Raphaël Deltombe, Philippe Moreau, Thomas Ingicco, Maxence Bigerelle and Julie Marteau which were incorrectly given as Borel Antony, Deltombe Raphaël, Moreau Philippe, Ingicco Thomas, Bigerelle Maxence and Marteau Julie.

The original Article has been corrected.

