# Supplementary material for: Optimization of use-wear detection and characterization on stone tool surfaces
Source: Sci Rep. 2021 Dec 17;11:24197. doi: 10.1038/s41598-021-03663-4 (PMC8683413; doi:10.1038/s41598-021-03663-4)
Supplement: Supplementary file 1 — Supplementary Information 1. [file 41598_2021_3663_MOESM1_ESM.pdf]

Supplementary Table S1

Reference	Acquisition technology and provider	Objectives (NA: numerical aperture)	Filter/Cut-off	Measurement area size	Metrological parameters
Dumont 1982[1]	Leitz "Mirau 2-beam system" interferometry	20x	-	575 µm (length of the photomicrograph)	Elevation change
Beyries et al. 1988[2]	Contact rugosimetry	NonApp	1 mm	256 lines x 256 points	Ra, Abbott curve
Kimball et al. 1995[3]	Atomic Force Microscopy	NonApp	-	15 x 15 µm	Peak, Valley, Ra
Anderson et al. 1998[4]	Optical rugosimetry + (contact rugosimetry, interferometry and AFM)	-	-	1024 x 1024 mm	Bearing area ratio, mean valley parameter
Stemp and Stemp 2001[5]	UBM (Ulrich Breitmeier Messtechnik) laser profilometry	-	-	4 or 6 mm	Rq at a series of length scales over the total scan distance, Hürst or roughening exponent H, fractal dimension (Dr = 2-H)
Astruc et al. 2003[6]	White light interference microscopy	-	Unfiltered	2.4 x 1.8 mm	Material volume, SPpm, SPvm, mean motif of waviness (W/AW) and slope roughness (R/AR), Rpm and Rvm
Stemp and Stemp 2003[7]	UBM laser profilometry	-	-	4 mm	Rq at a series of length scales over the total scan distance; Hürst or roughening exponent H; fractal dimension (Dr = 2-H)
Vargiolu et al. 2003[8]	Optical rugosimetry, mode Vertical-scanning interferometry (VSI)	20x	-	1.5 mm x 450 µm (?)	Material volume
Anderson et al. 2006[9]	Optical rugosimetry, mode Vertical-scanning interferometry (VSI)	20x	-	1.5 x 0.5 mm	Peak, Valley
Evans and Donahue 2008[10]	Laser scanning confocal microscopy (Olympus LEXT 3100)	100x (0.8NA)	-	3.8 x 3.8 µm<br>10.1 x 10.1 µm	Rq
Stemp et al. 2008[11]	UBM laser profilometry	-	-	2 mm long profile	Rq (versus evaluation length)
Stemp et al. 2009[12]	UBM laser profilometry	-	-	2 mm long profile	Relative lengths (RL, versus evaluation length)<br>Length-scale fractal analysis
Stemp et al. 2010[13]	UBM laser profilometry	-	-	2 mm long profile	Relative lengths (RL, versus evaluation length)<br>Length-scale fractal analysis
Stevens et al. 2010[14]	Laser scanning confocal microscopy (Olympus FluoView FV1000)	40x (0.6NA)	-	158 x 158 µm	11 variables: Rq, Rku, Rsk, FPO, MFOV, FAD, MRV, SA, Df, LAC, PLAC<br>3 variables finally selected: Mean resultant vector (MRV), surface area (SA), and fractal dimension (Df)
Astruc et al. 2011[15]	Confocal microscopy<br>Interferometry	-	-	900 x 900 µm	Spa, porosity and striation patterns
Evans et Macdonald 2011[16]	Laser scanning confocal microscopy (Olympus LEXT 3100)	100x (0.8NA)	-	3.8 x 3.8 µm<br>10.1 x 10.1 µm	Sq
	Focus variation microscopy (Alicona InfiniteFocus)<br>Laser scanning confocal microscopy (Olympus LEXT 4000)	50x (0.55NA)<br>50x (0.95NA)	Waviness/roughness cut-off value (lc = 3.24 mm)	12 x 12 µm	Sa, Sq
Faulks et al. 2011[17]	Atomic force microscopy	-	Flattened to correct for the tilt of the sample plane<br>Unfiltered	50 x 50 µm<br>2 x 2 µm	Ra, Skewness and Kurtosis on full measured area<br>Local Ra of the peaks, valleys, and transition regions
Procopiou et al. 2011[18]	Interferometry (phase detection)	-	-	2 x 2 mm	Local Normal, Sz, SMa, Multiscale Index Signature

Stemp et Chung 2011[19]	Laser scanning confocal microscopy (Olympus LEXT 4000)	20x	No substantial data filtering	643 x 643 µm	Scale-sensitive fractal analysis, RelA
Stemp et al. 2013[20]	Laser scanning confocal microscopy (Olympus LEXT 4000)	20x (0.6NA)	-	643 x 643 µm	Scale-sensitive fractal analysis, RelA
Evans et al. 2014[21]	Laser scanning confocal microscopy (Olympus LEXT 4000)	50x (0.95NA)<br>100x (0.95NA)	S-filter (Gaussian), 2.5 µm for antler, 1 µm for wood<br>L-filter (Gaussian), 50 µm for antler, 25 µm for wood	25 tiles of 50 x 50 µm per acquisition for antler<br>10 tiles of 25 x 25 µm per acquisition for wood	Sdr, S5v, Sda, Sdv, Sq, Sv, Sz, Sa, Smr, Smc, Sxp, Sdq, Vv, Vmc, Vvc
Ibáñez et al. 2014[22]	White light scanning confocal microscopy (Sensofar Plu Neox)	20x (0.45NA)	Robust Gaussian filter with 8 um cut-off	650 x 500 µm<br>100 x 100 µm	Sa, Sq, Sp, Sv, Sz, Sal, Sdc, maximum depth, mean depth and mean density of furrows
Macdonald 2014[23]	Focus variation microscopy (Alicona InfiniteFocus)	20x<br>50x	-	286 x 218 µm<br>10 x 10 µm	Sa, Sq
Macdonald and Evans 2014[24]	Laser scanning confocal microscopy (Olympus LEXT 4000)	20x<br>50x	-	30 x 30 µm	Sa
Stemp 2014[25]	See Stemp and Stemp 2001, 2003, Stemp et al. 2008, 2009, 2013 and Stemp and Chung 2011				
Key et al. 2015[26]	Laser scanning confocal microscopy (Olympus LEXT 4000)	20x (0.6NA)	Modal filter	643 x 643 µm	Srel, MSR
Stemp et al. 2015[27]	Laser scanning confocal microscopy (Olympus LEXT 4000)	20x (0.6NA)	Modal filter	643 x 300 µm	RelA, MSR
Stemp et al. 2015[28]	Laser scanning confocal microscopy (Olympus LEXT 4000)	20x (0.6NA)	Modal filter	643 x 643 µm	Srel, Asfc, MSR, SRC
Ibáñez et al. 2016[29]	White light scanning confocal microscopy (Sensofar Plu Neox)	20x (0.45NA)	Spatial filtering	Areas: 650 x 500 µm<br>Sub-areas: 200 x 200 µm	Sa, Sq, Sz, Sp, Sv, Sal, Str, Sdq, Sfd, MDF and mean density (MDenF)
Macdonald et al. 2018[30]	Laser scanning confocal microscopy (Olympus LEXT 4000)	50x	Leveled<br>F operator (polynomial of degree 3)<br>Robust Gaussian filter with cut-off of 25 µm	-	Sq
Stemp et al. 2018[31]	Laser scanning confocal microscopy (Olympus LEXT 4000)	20x (0.6NA)	-	643 x 643 µm	Srel, Asfc, SRC
Calandra et al. 2019[32]	Laser scanning confocal microscopy (Zeiss LSM 800 MAT)	50x (0.75NA)<br>50x (0.95NA)	Gaussian microroughness low-pass filter (λs = 2.5 µm)<br>Gaussian roughness high-pass filter (λc = 0.8 mm, end effects not managed)	255.56 x 255.56 µm	Ra, Sa, Sal, Sdq, Sdr, Sku, Sk, Smc, Smq, Smr, Smr1, Smr2, Sp, Spk, Spq, Sq, Ssk, Std, Str, Sv, Svk, Svq, Sxp, Sz, Vm, Vmc, Vmp, Vv, Vvc, Vvv
Calandra et al. 2019[33]	Laser scanning confocal microscopy (Zeiss LSM 800 MAT)<br><br>3D optical profilometry (Sensofar S-Neox)	LSM800: 50x (0.95NA)<br><br>S-Neox: 50x (0.3NA)	Gaussian low-pass S-filter<br>F operator (polynomial of degree 3)<br>Gaussian high-pass L-filter<br>Setting threshold the surface between 0.010 and 99.9% material ratio to remove the aberrant positive and negative spikes	255.56 x 255.56 µm (LSM800)<br><br>350.88 x 264.19 µm (S neox)	Smc, Smr, Sxp, Sa, Sku, Sp, Sq, Ssk, Sv, Sz, Sal, Std, Str, Sk, Smq, Smr1, Smr2, Spk, Spq, Svk, Svq, Vm, Vmc, Vmp, Vv, Vvc, Vvv
Ibáñez et al. 2019[34]	White light scanning confocal microscopy (Sensofar Plu Neox)	20x (0.45NA)	Leveling: least squares (LS) plane method<br>Spatial filtering<br>The texture, which is the surface measured in the analysis, is calculated by subtracting the filtered surface from the source surface	Areas: 650 x 500 µm<br>Subareas: 50 x 50 µm	Sq, Sz, Sp, Sv, Sal, Str, Sdq, Sds, S5p, Spc, Spd, Sdc, Sbi, Sci, Svi, MDF, MDenF
Macdonald et al. 2019[35]	Scanning microdisplay confocal microscopy (Sensofar S-Neox)	50x (0.8NA)	Leveling by rotation using the least square plane<br>F operator (polynomial of degree 2)<br>Robust Gaussian filter with a roughness cut-off of 25 µm	340 x 284 µm	Sq, Ssk, Sku, Sp, Sv, Sz, Sa

Pfleging et al. 2019[36]	Focus variation microscopy<br>(Alicona InfiniteFocus G4)	50x (0.55NA)<br>100x (0.8NA)	Form removal by removing a fitted 2D plane from the original data<br>Gaussian filter applied with a SD of 6 µm for 50x images and 4 µm for 100x images<br>Subtraction of the filtered image from the original image<br>Median filter applied using a squared kernel with side length of 20 pixels<br>All of the resulting pixel values, which exceeded a threshold of 1 µm, were classified as outliers and were discarded	50x: 300 x 200 µm<br>100x: 140 x 100 µm	Sa, Sq, Ssk, Sku, relA, D (fractal dimension), critical scale
Álvarez-Fernández et al. 2020[37]	Laser scanning confocal microscopy<br>(Olympus LEXT 3000)	20x (0.45NA)	Smoothing function to eliminate random noise<br>Surface correction filter to correct the natural curvature of the tools<br>Cut-off value (λc 13.3 µm)	Areas: 640 x 480 µm<br>Subareas: 55 x 42 µm	Rp, Rv, Rz, Rc, Ra, Rq, RzJIS
Pedergnana et al. 2020[38]	Laser scanning confocal microscopy<br>(Olympus LEXT 4000)	50x (0.95NA)	Leveling: least squares (LS) plane by subtraction<br>F operator (polynomial of degree 3)<br>Clean the sub-areas from defects by removing outliers and thresholding<br>Robust Gaussian filter with cut-off of 2.5 µm	Areas: 256 x 256 µm<br>Subareas: 50 x 50 µm	ISO 25178-2, scale-sensitive fractal analysis, furrow analysis, texture isotropy and texture direction = 33 parameters
Zupancich and Cristiani 2020[39]	Digital stereomicroscopy<br>(Zeiss AxioZoom V16 ?)	50x	Leveling<br>F-Operator (polynomial of degree 2)<br>Robust Gaussian filter with cut-off of 25 µm	2 x 2.5 mm	Sq, Sv
Chondrou et al. 2021[40]	Confocal rugosimeter	(?) laser sensor of 2,5 mm<br>(?) laser sensor of 400 µm	-	Macro-scale: 14 x 14 mm<br>Micro-scale: 500 x 500 µm	SMa
Ibáñez and Mazzucco 2021[41]	White light scanning confocal microscopy<br>(Sensofar Plu Neox)	20x (0.45NA)	Leveling: least squares (LS) plane method<br>Spatial filtering (arithmetic mean operator consisting in averaging each point with its 13 × 13 neighboring points)<br>The texture, which is the surface measured in the analysis, is calculated by subtracting the filtered surface from the source surface	50 x 50 µm	22 parameters from ISO 25178: Sq, Sv, Smc, Str, Sdq, Sdr, Vm, Vvv, Spd, Spc, S10z, S5p, Sda, Sdv, Shv, Sk, Svk, Smr1, Smr2, Sds, Smean, Stdi
Paixão et al. 2021[42]	Laser scanning confocal microscopy<br>(Zeiss LSM 800 MAT)	50x (0.75NA)	Leveling: least squares (LS) plane method<br>F operator (polynomial of degree 3)<br>Low-pass filter (λs = 2.5 µm)	255.6 x 255.6 µm	Sa, Sq, Ssk, Sku, Sp, Sv, Sz, Sal, Str, Std, Sdq, Sdr, Smr, Smc, Sdc, Sxp, Vm, Vv, Vmp, Vmc, Vvc, Vvv, Spd, Spc, S10z, S5p, S5v, Sda, Sha, Sdv, Shv, madf, metf, medf, Tr, Tr1R, Tr2R, Tr3R, IsT, Asfc, Smfc, HAsfc9, HAsfc81, epLsar, NewEplsar
Pichon et al. 2021[43]	White light scanning confocal microscopy<br>(Sensofar Plu Neox)	20x (0.45NA)	Leveling: least squares (LS) plane method<br>The polish texture was isolated from irregularities of the flint topography by moving a small filtering matrix over the surface and by subtracting the filtered surface from the source surface	Areas: 650 x 500 µm<br>Subareas: 200 x 200 µm	Sa, Sq, Sz, Sp, Sv, Sal, Str, Sdq, Sfd, MDF and mean density (MDenF)

Rodriguez et al. 2021[44]	3D optical profilometry (Sensofar S-Neox)	20× (0.45NA)<br>50× (0.80NA)	Gaussian low-pass S-filter (S1 nesting index = 1.093 µm for the 20× objective and 0.437 µm for the 50×, end effects managed)<br>F operator (polynomial of degree 3)<br>Gaussian high-pass L-filter (L nesting index = 327.980 µm for the 20× objective and 131.200 µm for the 50×, end effects managed)<br>Setting threshold surface between 0.010 and 99.9% material ratio to remove the aberrant positive and negative spikes	20×: 872.68µm x 655.965µm<br>50×: 350.88µm × 264.19µm	Sa, Sal, Spc and Smr1

Supplementary Table S1a: review of acquisition technologies and providers, objectives, filters, measurement area sizes and metrological parameters used in quantitative use-wear analyses since the 80's.

Reference	Number of measurements	Number of measured artefacts	Tool raw material	Experimentally worked material
Dumont 1982[1]	7	1 archaeological	Flint	Wood
Beyries et al. 1988[2]	-	5 (at least) experimental	Flint	Cereals, bone, hide + unused
Kimball et al. 1995[3]	38	2 experimental	Flint	Wood, antler, dry hide, meat + unused
Anderson et al. 1998[4]	-	10 experimental, 2 ethnographical and 6 archaeological	Flint, obsidian	Cereals, reed, straw + unused
Stemp and Stemp 2001[5]	-	5(?) experimental	Chert, obsidian	Antler, shell, pottery + unused
Astruc et al. 2003[6]	4(?)	2(?) experimental	Chert	Picrolite, diabase
Stemp and Stemp 2003[7]	22(?)	7(?) experimental	Flint	Wood, pottery + unused
Vargiolu et al. 2003[8]	-	3 experimental	Flint	Straw + unused
Anderson et al. 2006[9]	1	1 experimental	Flint	Straw + unused
Evans and Donahue 2008[10]	2 scales * 10 areas * 12 artefacts = 240	12 experimental	Flint	Antler, wood, fresh hide, dry hide, greasy hide + unused
Stemp et al. 2008[11]	11 to 25 profiles on each tool	8 experimental	Flint	Soaked antler, dry cow hide, sugar mapple wood, queen conch shell, dry antler
Stemp et al. 2009[12]	11 to 25 profiles on each tool	4 experimental	Flint	Soaked antler, dry cow hide, sugar mapple wood, queen conch shell
Stemp et al. 2010[13]	48 or 64 profiles	4 experimental	Flint	Queen conch shell, dry antler
Stevens et al. 2010[14]	15 areas of 10 µm² on each image	Training set: 36 experimental for polish analysis and 48 for edge damage analysis (the 36 from polish analysis included)<br>Test set: 20 experimental from training set and 16 archaeological	Chert	Antler, wood, soft plants, dry hide, meat + unused
Astruc et al. 2011[15]	11	1 archaeological	Obsidian	-
Evans et Macdonald 2011[16]	20	1 experimental and 1 archaeological	Flint	Greasy hide
	2	1 experimental	Flint	Antler
Faulks et al. 2011[17]	60	5 archaeological	Flint	-
Procopiou et al. 2011[18]	17(?)	17 experimental	Diasporite	Sandstone, granite, emery
Stemp et Chung 2011[19]	3 areas*2 zones*3 artefacts = 18	3 experimental	Obsidian	Dry cow hide, maple wood, queen conch shell
Stemp et al. 2013[20]	48	4 experimental	Quartzite	Dry hide, fresh hide + unused
Evans et al. 2014[21]	5 (125 tiles) for antler x 4 use-duration interval<br>5 (50 tiles) for wood x 4 use-duration interval<br>= 40 (700 tiles)	4 experimental	Flint	Soaked antler, wood
Ibáñez et al. 2014[22]	92 (703 subareas)	7 experimental and 4 archaeological	Flint	Wild cereals, domestic cereals + unused
Macdonald 2014[23]	5 areas x 15 subareas x 8 tools = 600	8 experimental	Chert	Antler, wood, dry hide, meat, wheat + unused
Macdonald and Evans 2014[24]	5 areas x 3 cleaning stage x 2 tools = 30	2 experimental	Chert	Wheat
Stemp 2014[25]	See Stemp and Stemp 2001, 2003, Stemp et al. 2008, 2009, 2013 and Stemp and Chung 2011			
Key et al. 2015[26]	7 per tool = 196	28 experimental	Basalt	Wood (Oak)
Stemp et al. 2015[27]	6 per tool before and after use = 36	3 experimental	Obsidian	Meat (raw beef)
Stemp et al. 2015[28]	7 per tool	23 experimental	Basalt	English oak
Ibáñez et al. 2016[29]	4 to 8 sample areas on each tool and 12 to 45 subsamples per tool	20 experimental and 68 archaeological	Flint	Spontaneously-growing wild cereals, cultivated wild cereals, domestic cereals, reeds
Macdonald et al. 2018[30]	5 per cast = 45	3 casts per object = 9 casts	British penny (copper-plated steel), tooth, chert, Huntsman low viscosity Araldite DBF epoxy casting resin	Chert was used on antler
Stemp et al. 2018[31]	12 per tool = 48	4 experimental	Quartzite	Fresh and dry hide + unused

Calandra et al. 2019[32]	3 per tool measured 3 times with each objective	2 experimental	Flint, metaquartzite	Dry wood, giant cane stem
Calandra et al. 2019[33]	10 per tool with each microscope = 40	2 experimental	Flint	Dry wood
Ibáñez et al. 2019[34]	6 sample areas per tool and 12 to 76 subsamples per tool	30 experimental	Flint	Bone, antler, wood, fresh hid, dry hide, wild cereals, domestic cereals, reed
Macdonald et al. 2019[35]	10 per tool for sediment wear = 40<br>5 per tool when possible, for hafting wear = 16<br>20 on the unused hoe	5 experimental	Oolitic dolomite	Sediment + unused<br>Hafted with fresh paper mulberry and hemp twine
Pfleging et al. 2019[36]	162 for Sa, Sq, Ssk and Sku with 50x<br>262 for Sa, Sq, Ssk and Sku with 100x<br>302 for fractal dimension and critical scale<br>60 for 110N<br>39 for 90N<br>39 for 60N<br>32 for 30N<br>(224 measurements indicated in online resource)	48 experimental (?) (From Flake ID in online resource)	Flint	Wood
Álvarez-Fernández et al. 2020[37]	13 areas<br>50 subareas on worn surfaces + 50 subareas on unworn surfaces	11 experimental	Chert	Wood, butchery + unused
Pedergnana et al. 2020[38]	43 scans<br>2 subareas per scan = 86 or 78 ("full dataset") and 70 ("restricted dataset")	14 experimental	Quartzite	Wood, bone, soaked antler, fresh and dry skin, cane + unused
Zupancich and Cristiani 2020[39]	4 per tool = 36	9 experimental	Sandstone	Bone, tendon, fresh hide, dry hide, acorn, wild grass grain, oat, mixed marrow and ochre
Chondrou et al. 2021[40]	Macro-scale: 1 per tool<br>Micro-scale: 3 per tool	24 experimental<br>32 archaeological	Experimental: sandstone, andesite, granite<br>Archaeological: basalt, sandstone, greenschist, gneiss, schist, conglomerate, amphibolite	Dehusked einkorn, hulled einkorn, dehusked millet, hulled millet, barley (malt), untreated bitter vetch, split, sieved and winnowed bitter vetch, untreated linseed, roasted linseed, dried acorns, roasted acorns, untreated lentils, split, sieved and winnowed lentils, untreated poppy seeds, roasted poppy seeds, unripe and smoked spelt, untreated grass pea, split, sieved and winnowed grass pea,
Ibáñez and Mazzucco 2021[41]	16 x 6 steps of use x 12 used zones = 1152<br>117 on unused surfaces<br>12 used zones x 8 areas x 7 steps of use = 672	10 experimental	Flint	Soaked antler, dry hide, fresh reed, fresh bone, dry wood (pine), cured meat (pig) + unused
Paixão et al. 2021[42]	3 per tool (when possible)	31 experimental	Limestone	Fresh bone (heck cattle femurs), flint, humid acorn, dry acorn
Pichon et al. 2021[43]	6 to 10 areas per tool<br>3 to 4 subareas per areas	48 archaeological and 19 experimental from Ibañez et al., 2016	Flint	Spontaneously-growing wild cereals, cultivated wild cereals, domestic cereals, reeds
Rodriguez et al. 2021[44]	3 to 5 measurements of different areas on each sample<br>Antler = 10, Bone = 10, Beech wood = 5, Ivory = 5, Spruce wood = 5, Raw = 40<br>Total of 75 measurements	7 experimental	Flint	Dry bone, ivory, antler and wood (beechwood and spruce) + unused

Supplementary Table S1b: review of the number of measurements and measured artefacts of the raw material of the stone tool and worked material used in quantitative use-wear analyses since the 80's.

References

1. Dumont, J. The Quantification of Microwear Traces: A New Use for Interferometry. *World Archaeology* **14**, 206–217 (1982).
2. Beyries, S., Delamare, F. & Quantin, J.-C. Tracéologie et rugosimétrie tridimensionelle. in *Industries Lithiques: Tracéologie et Technologie vol 2* (ed. Beyries, S.) 115–132 (Archeopress, 1988).
3. Kimball, L. R., Kimball, J. F. & Allen, P. E. Microwear polishes as viewed through the atomic force microscope. *Lithic Technology* **20**, 6–28 (1995).
4. Anderson, P. C., Astruc, L., Vargiolu, R. & Zahouani, H. Contribution of quantitative analysis of surface states to a multi-method approach for characterizing plant-processing traces on flint tools with gloss. in *Functional Analysis of Lithic Artefacts: Current State of Research, Proceedings of the XIII Congress of the International Union of Prehistoric and Protohistoric Sciences. Volume 6 - Tome II* (ed. Alhaique, F.) 1151–1160 (A.B.A.C.O., 1998).
5. Stemp, W. J. & Stemp, M. UBM Laser Profilometry and Lithic Use-Wear Analysis: A Variable Length Scale Investigation of Surface Topography. *Journal of Archaeological Science* **28**, 81–88 (2001).
6. Astruc, L., Vargiolu, R. & Zahouani, H. Wear assessments of prehistoric instruments. *Wear* **255**, 341–347 (2003).
7. Stemp, W. J. & Stemp, M. Documenting Stages of Polish Development on Experimental Stone Tools: Surface Characterization by Fractal Geometry Using UBM Laser Profilometry. *Journal of Archaeological Science* **30**, 287–296 (2003).
8. Vargiolu, R., Zahouani, H. & Anderson, P. C. Etude tribologique du processus d’usure des lames de silex et fonctionnement du tribulum. in *Le traitement des récoltes: un regard sur la diversité du Néolithique au présent* (eds. Anderson, P. C., Cummings, L. S., Schippers, T. K. & Simonel, B.) 439–454 (2003).
9. Anderson, P. C., Georges, J.-M., Vargiolu, R. & Zahouani, H. Insights from a tribological analysis of the tribulum. *Journal of Archaeological Science* **33**, 1559–1568 (2006).
10. Evans, A. A. & Donahue, R. E. Laser scanning confocal microscopy: a potential technique for the study of lithic microwear. *Journal of Archaeological Science* **35**, 2223–2230 (2008).

11. Stemp, W. J., Childs, B. E., Vionnet, S. & Brown, C. A. The Quantification of Microwear on Chipped Stone Tools: Assessing the Effectiveness of Root Mean Square Roughness (Rq). *Lithic Technology* **33**, 173–189 (2008).
12. Stemp, W. J., Childs, B. E., Vionnet, S. & Brown, C. A. Quantification and Discrimination of Lithic Use-Wear: Surface Profile Measurements and Length-Scale Fractal Analysis. *Archaeometry* **51**, 366–382 (2009).
13. Stemp, W. J., Childs, B. E. & Vionnet, S. Laser profilometry and length-scale analysis of stone tools: second series experiment results. *Scanning* **32**, 233–243 (2010).
14. Stevens, N. E., Harro, D. R. & Hicklin, A. Practical quantitative lithic use-wear analysis using multiple classifiers. *Journal of Archaeological Science* **37**, 2671–2678 (2010).
15. Astruc, L. *et al.* Multi-scale tribological analysis of the technique of manufacture of an obsidian bracelet from Aşıklı Höyük (Aceramic Neolithic, Central Anatolia). *Journal of Archaeological Science* **38**, 3415–3424 (2011).
16. Evans, A. A. & Macdonald, D. Using metrology in early prehistoric stone tool research: further work and a brief instrument comparison. *Scanning* **33**, 294–303 (2011).
17. Faulks, N. R., Kimball, L. R., Hidjrati, N. & Coffey, T. S. Atomic force microscopy of microwear traces on Mousterian tools from Myshtylagty Lagat (Weasel Cave), Russia. *Scanning* **33**, 304–315 (2011).
18. Procopiou, H., Boleti, A., Vargiolu, R. & Zahouani, H. The role of tactile perception during stone-polishing in Aegean prehistory (5th–4th millennium B.C.). *Wear* **271**, 2525–2530 (2011).
19. Stemp, W. J. & Chung, S. Discrimination of surface wear on obsidian tools using LSCM and RelA: pilot study results (area-scale analysis of obsidian tool surfaces). *Scanning* **33**, 279–293 (2011).
20. Stemp, W. J., Lerner, H. J. & Kristant, E. H. Quantifying Microwear on Experimental Mistassini Quartzite Scrapers: Preliminary Results of Exploratory Research Using LSCM and Scale-Sensitive Fractal Analysis. *Scanning* **35**, 28–39 (2013).
21. Evans, A. A., Macdonald, D. A., Giusca, C. L. & Leach, R. K. New method development in prehistoric stone tool research: Evaluating use duration and data analysis protocols. *Micron* **65**, 69–75 (2014).
22. Ibáñez, J. J., González-Urquijo, J. E. & Gibaja, J. Discriminating wild vs domestic cereal harvesting micropolish through laser confocal microscopy. *Journal of Archaeological Science* **48**, 96–103 (2014).

23. Macdonald, D. A. The application of focus variation microscopy for lithic use-wear quantification. *Journal of Archaeological Science* **48**, 26–33 (2014).
24. Macdonald, D. A. & Evans, A. A. Evaluating Surface Cleaning Techniques of Stone Tools Using Laser Scanning Confocal Microscopy. *Microscopy Today* **22**, 22–27 (2014).
25. Stemp, W. J. A review of quantification of lithic use-wear using laser profilometry: a method based on metrology and fractal analysis. *Journal of Archaeological Science* **48**, 15–25 (2014).
26. Key, A. J. M., Stemp, W. J., Morozov, M., Proffitt, T. & de la Torre, I. Is Loading a Significantly Influential Factor in the Development of Lithic Microwear? An Experimental Test Using LSCM on Basalt from Olduvai Gorge. *J Archaeol Method Theory* **22**, 1193–1214 (2015).
27. Stemp, W. J., Andruskiewicz, M. D., Gleason, M. A. & Rashid, Y. H. Experiments in ancient Maya bloodletting: quantification of surface wear on obsidian blades. *Archaeol Anthropol Sci* **7**, 423–439 (2015).
28. Stemp, W. J., Morozov, M. & Key, A. J. M. Quantifying lithic microwear with load variation on experimental basalt flakes using LSCM and area-scale fractal complexity (Asfc). *Surf. Topogr.: Metrol. Prop.* **3**, 034006 (2015).
29. Ibáñez, J. J., Anderson, P. C., González-Urquijo, J. & Gibaja, J. Cereal cultivation and domestication as shown by microtexture analysis of sickle gloss through confocal microscopy. *Journal of Archaeological Science* **73**, 62–81 (2016).
30. Macdonald, D. A., Harman, R. & Evans, A. A. Replicating surface texture: Preliminary testing of molding compound accuracy for surface measurements. *Journal of Archaeological Science: Reports* **18**, 839–846 (2018).
31. Stemp, W. J., Lerner, H. J. & Kristant, E. H. Testing Area-Scale Fractal Complexity (Asfc) and Laser Scanning Confocal Microscopy (LSCM) to Document and Discriminate Microwear on Experimental Quartzite Scrapers. *Archaeometry* **60**, 660–677 (2018).
32. Calandra, I. *et al.* The effect of numerical aperture on quantitative use-wear studies and its implication on reproducibility. *Sci Rep* **9**, 1–10 (2019).
33. Calandra, I. *et al.* Back to the edge: relative coordinate system for use-wear analysis. *Archaeol Anthropol Sci* (2019) doi:10.1007/s12520-019-00801-y.

34. Ibáñez, J. J., Lazuen, T. & González-Urquijo, J. Identifying Experimental Tool Use Through Confocal Microscopy. *J Archaeol Method Theory* **26**, 1176–1215 (2019).

35. Macdonald, D. A., Xie, L. & Gallo, T. Here's the dirt: First applications of confocal microscopy for quantifying microwear on experimental ground stone earth working tools. *Journal of Archaeological Science: Reports* **26**, 101861 (2019).

36. Pfleging, J., Iovita, R. & Buchli, J. Influence of force and duration on stone tool wear: results from experiments with a force-controlled robot. *Archaeol Anthropol Sci* **11**, 5921–5935 (2019).

37. Álvarez-Fernández, A., García-González, R., Márquez, B., Carretero, J. M. & Arsuaga, J. L. Butchering or wood? A LSCM analysis to distinguish use-wear on stone tools. *Journal of Archaeological Science: Reports* **31**, 102377 (2020).

38. Pedergnana, A. *et al.* Polish is quantitatively different on quartzite flakes used on different worked materials. *PLOS ONE* **15**, e0243295 (2020).

39. Zupancich, A. & Cristiani, E. Functional analysis of sandstone ground stone tools: arguments for a qualitative and quantitative synergetic approach. *Sci Rep* **10**, 15740 (2020).

40. Chondrou, D. *et al.* How do you like your cereal? A qualitative and quantitative use-wear analysis on archaeological grinding tools from prehistoric Greek sites. *Wear* **476**, 203636 (2021).

41. Ibáñez, J. J. & Mazzucco, N. Quantitative use-wear analysis of stone tools: Measuring how the intensity of use affects the identification of the worked material. *PLOS ONE* **16**, e0257266 (2021).

42. Paixão, E. *et al.* Using mechanical experiments to study ground stone tool use: Exploring the formation of percussive and grinding wear traces on limestone tools. *Journal of Archaeological Science: Reports* **37**, 102971 (2021).

43. Pichon, F., Ibáñez-Estevez, J. J., Anderson, P. C., Douché, C. & Coqueugniot, É. Harvesting cereals at Dja'de el-Mughara in the northern Levant: New results through microtexture analysis of Early PPNB sickle gloss (11th millennium cal BP). *Journal of Archaeological Science: Reports* **36**, 102807 (2021).

44. Rodriguez, A. *et al.* The effect of worked material hardness on stone tool wear. *OSF Preprints* (2021) doi:10.31219/osf.io/uhkbr.
