# Supplementary material for: Optimization of use-wear detection and characterization on stone tool surfaces
Source: Sci Rep. 2021 Dec 17;11:24197. doi: 10.1038/s41598-021-03663-4 (PMC8683413; doi:10.1038/s41598-021-03663-4)
Supplement: Supplementary file 3 — Supplementary Information 3. [file 41598_2021_3663_MOESM3_ESM.pdf]

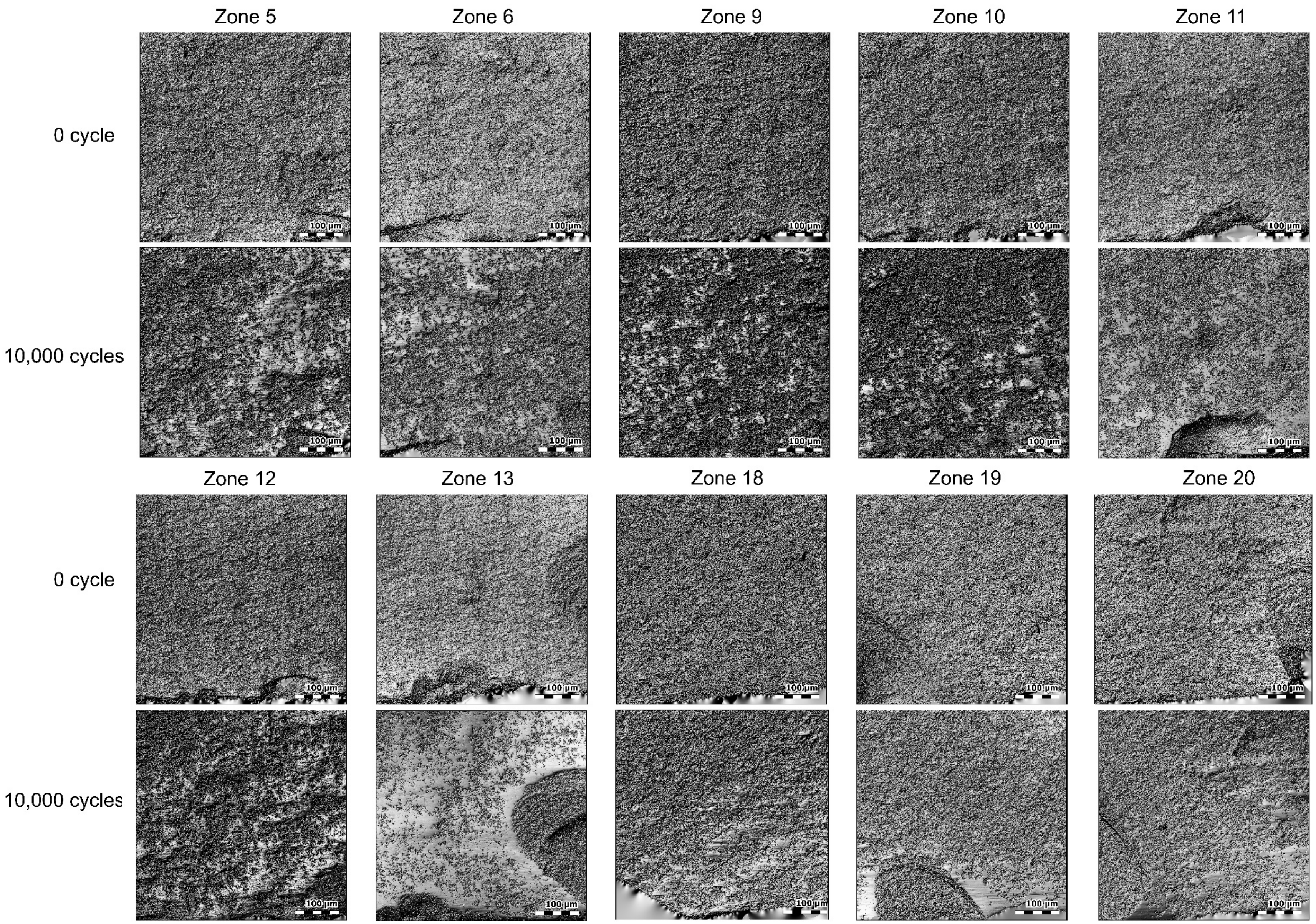

Supplementary Figure S3: locations visually showing the most extensive wear on the edge of the used flint tool.
