# Supplementary material for: Optimization of use-wear detection and characterization on stone tool surfaces
Source: Sci Rep. 2021 Dec 17;11:24197. doi: 10.1038/s41598-021-03663-4 (PMC8683413; doi:10.1038/s41598-021-03663-4)
Supplement: Supplementary file 5 — Supplementary Information 5. [file 41598_2021_3663_MOESM5_ESM.pdf]

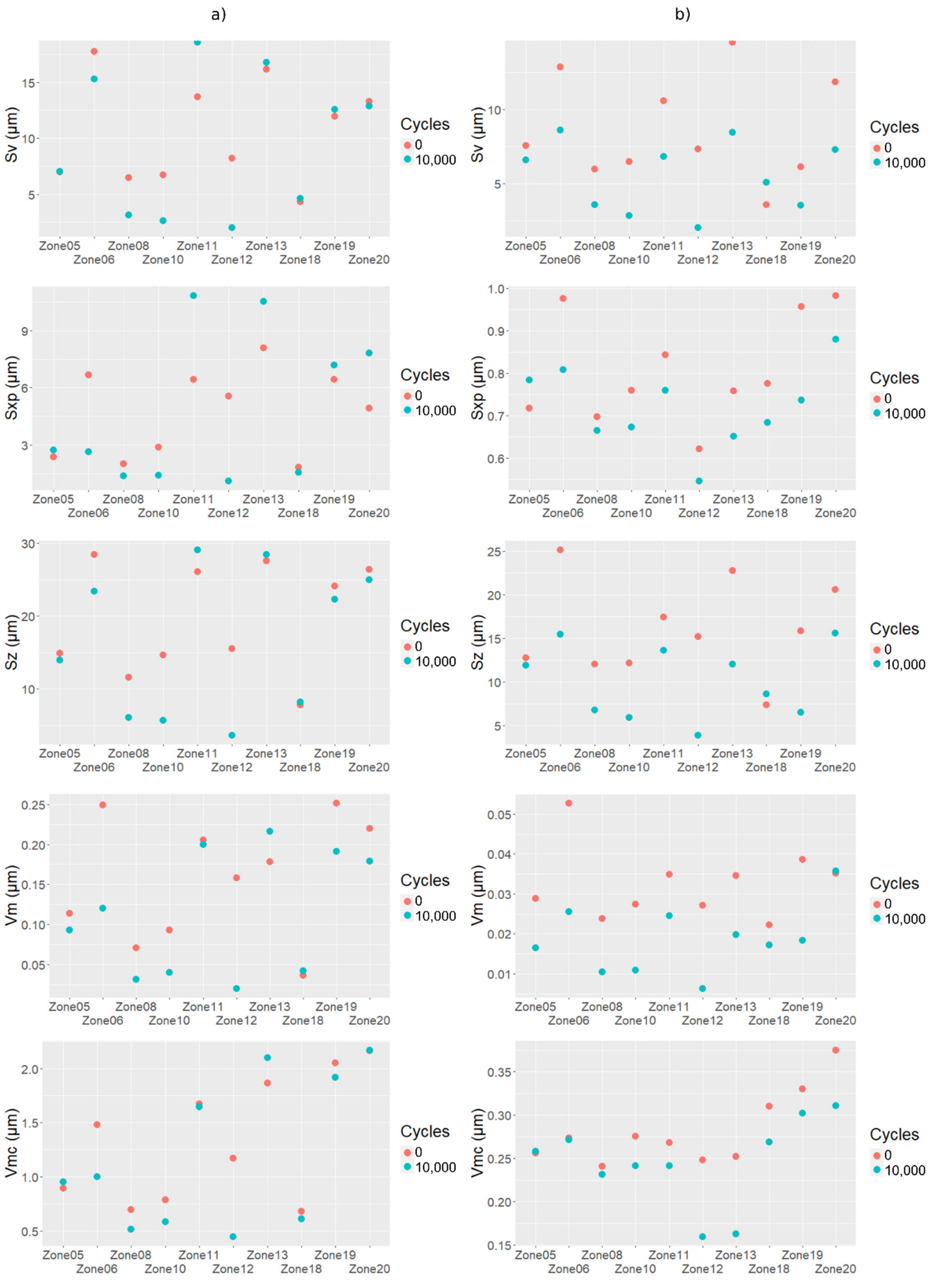

Supplementary Figure S5a: maximum pit depth (Sv), peak extreme height (Sxp), maximum height (Sz), material volume (Vm) and core material volume (Vmc) computed for the 10 selected measurements showing the largest wear traces (a) without filtering, (b) with filtering (high-pass filter with a 25 µm cut-off length).

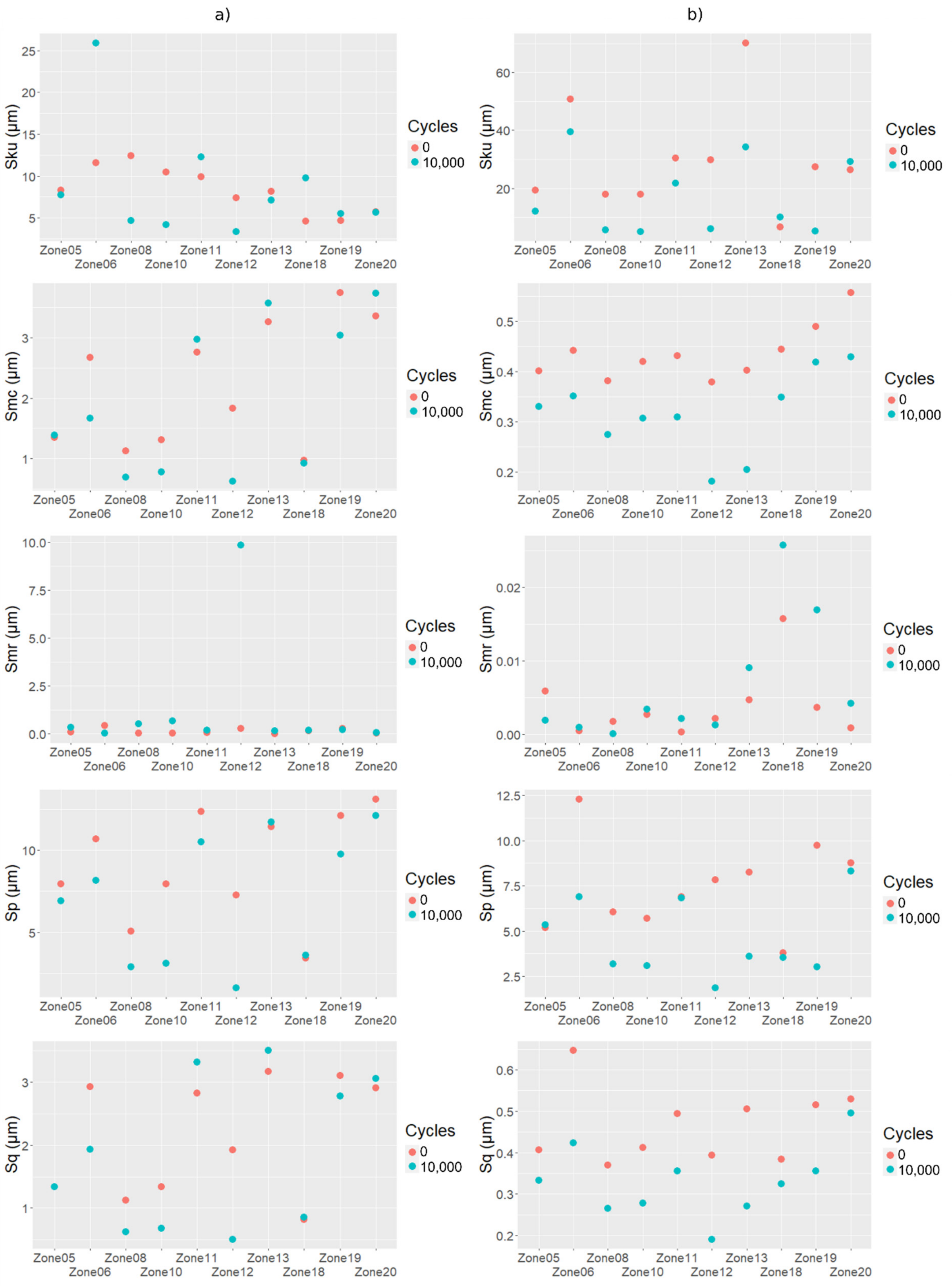

Supplementary Figure S5b: kurtosis (Sku), inverse areal material ratio (Smc), peak material portion (Smr), maximum peak height (Sp) and root mean square height (Sq) computed for the 10 selected measurements showing the largest wear traces (a) without filtering, (b) with filtering (high-pass filter with a 25 µm cut-off length).

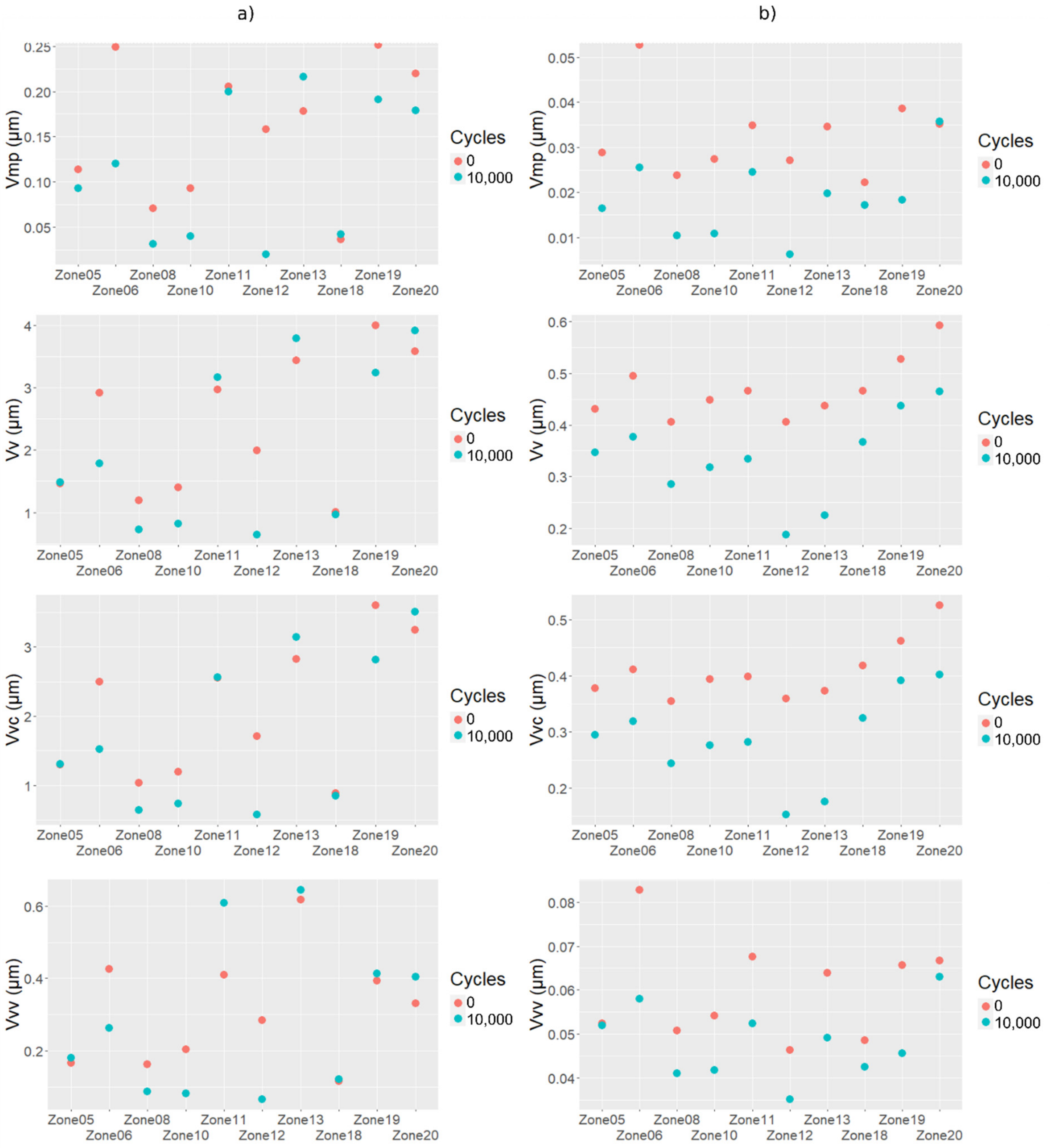

Supplementary Figure S5c: peak material volume (Vmp), void volume (Vv), core void volume (Vvc) and dale void volume (Vvv) computed for the 10 selected measurements showing the largest wear traces (a) without filtering, (b) with filtering (high-pass filter with a 25 µm cut-off length).
