# Supplementary material for: Optimization of use-wear detection and characterization on stone tool surfaces
Source: Sci Rep. 2021 Dec 17;11:24197. doi: 10.1038/s41598-021-03663-4 (PMC8683413; doi:10.1038/s41598-021-03663-4)
Supplement: Supplementary file 7 — Supplementary Information 7. [file 41598_2021_3663_MOESM7_ESM.pdf]

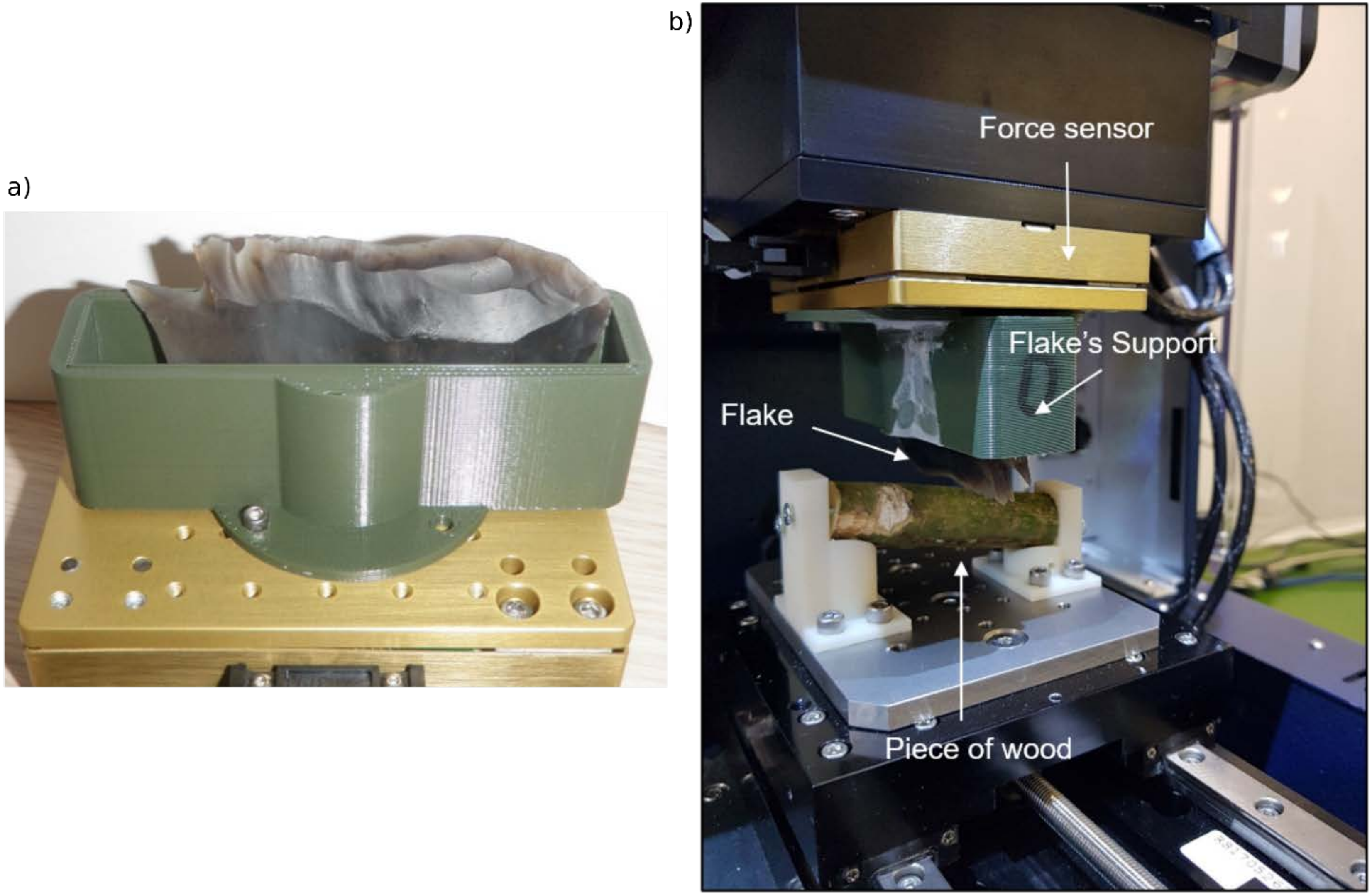

Supplementary Figure S7: Used flake mounted in a specially designed support (a) and installed in the tribometer Bruker UMT TriboLab (b).
